# The Role of Glymphatic System in Alzheimer’s and Parkinson’s Disease Pathogenesis

**DOI:** 10.3390/biomedicines10092261

**Published:** 2022-09-13

**Authors:** Francesca R. Buccellato, Marianna D’Anca, Maria Serpente, Andrea Arighi, Daniela Galimberti

**Affiliations:** 1 Department of Biomedical, Surgical and Dental Sciences, University of Milan, 20122 Milan, Italy; 2 Neurodegenerative Diseases Unit, Fondazione IRCCS Ca’ Granda, Ospedale Maggiore Policlinico, 20122 Milan, Italy

**Keywords:** Alzheimer’s disease, Parkinson’s disease, glymphatic system, Aquaporin 4, neuroimaging

## Abstract

Alzheimer’s disease (AD) is the most common cause of neurodegenerative dementia, whilst Parkinson’s disease (PD) is a neurodegenerative movement disorder. These two neurodegenerative disorders share the accumulation of toxic proteins as a pathological hallmark. The lack of definitive disease-modifying treatments for these neurogenerative diseases has led to the hypothesis of new pathogenic mechanisms to target and design new potential therapeutic approaches. The recent observation that the glymphatic system is supposed to be responsible for the movement of cerebrospinal fluid into the brain and clearance of metabolic waste has led to study its involvement in the pathogenesis of these classic proteinopathies. Aquaporin-4 (AQP4), a water channel located in the endfeet of astrocyte membrane, is considered a primary driver of the glymphatic clearance system, and defective AQP4-mediated glymphatic drainage has been linked to proteinopathies. The objective of the present review is to present the recent body of knowledge that links the glymphatic system to the pathogenesis of AD and PD disease and other lifestyle factors such as sleep deprivation and exercise that may influence glymphatic system function. We will also focus on the potential neuroimaging approaches that could identify a neuroimaging marker to detect glymphatic system changes.

## 1. Introduction

The knowledge of the molecular mechanisms behind water transport in the brain has been recently expanded by the rising concept of a new “glymphatic system”. Virchow–Robin spaces are fluid-filled spaces that permit the flux of cerebrospinal fluid (CSF) into the brain parenchyma. These spaces are also known as perivascular spaces (PVSs) of the central nervous system (CNS) and play a significant role in managing the influx of a large amount of the subarachnoid CSF through the brain parenchyma and efflux of brain interstitial fluid (ISF) before being cleared via perivenous pathways [1,2]. This clearance network is referred to as the “glymphatic system”. The term is also coined considering that astrocytes carrying the aquaporin-4 (AQP-4) water channel support the CSF-ISF exchange and solute clearance [1,2].

Even though the existence of the glymphatic system and the role of AQP4 in the homeostasis of the brain water have been recently questioned, the recent literature focuses on the role of lymphatic and glymphatic pathways in facilitating extracellular solutes’ clearance, including soluble amyloid-β (Aβ) and α-synuclein, from the brain [2,3,4,5,6,7,8]. PVSs increased or dilated, and disruption of the blood-brain barrier (BBB) may impact the transport of protein and toxin to the periphery and have been linked to dementia in progressive neurodegenerative disorders such as Alzheimer’s disease (AD) and Parkinson’s diseases (PD) [7,8].

Consequently, alterations in brain lymphatic and glymphatic system drainage function may contribute to the failure of toxic proteins clearance in neurodegenerative diseases like AD and PD [7,8,9,10]. Dementia can also be considered a common tract in the two diseases as most patients exhibit cognitive disfunction during PD. PD patients with dementia (PDD) also show amyloid- plaques and tau neurofibrillary tangles [9,10,11]. In the present review, we will analyze the recent literature regarding the impaired function of glymphatic system and its role in the pathogenesis of AD and PD.

A method to measure glymphatic pathway function may be considered an early approach in asymptomatic or diagnosed patients to evaluate disease susceptibility and progression [12]. To this aim, neuroimaging procedures can evaluate the brain drainage system and correlate compromised glymphatic function to reduced cognitive performance [13]. In this view glymphatic system may represent a potential therapeutic target in both PD and AD.

The observation that fluid clearance in brain parenchyma is impaired in AQP-4 knockout mice highlight the potential role of AQP4 as a major driver in brain fluid homeostasis [4]. In AQP4 knockout mice, it is observed that the loss of AQP4 eliminates the difference in day–night glymphatic drainage. Sleep deprivation can be linked to increases in beta-amyloid accumulation, in ISF tau and CSF tau and α-synuclein in humans, demonstrating that alteration in circadian rhythms can be considered risk factors in aging-related neurodegenerative diseases [14,15,16,17]. Further, decreased expression of AQP4 is described both in patients and in animal models of AD and PD [18,19]. Even though the involvement of AQP-4 in the pathophysiology of neurodegenerative disease is still controversial and unclear, the studies on animal models in this field can help understand the molecular aspects of the pathological changes in the brain drainage system and will be analyzed in the present review [19]. Recently, the scientific community has opened to the possibility that inhibitors of AQP4 could be considered a therapeutic approach in the treatment of neurological diseases [20,21].

Neurodegenerative diseases are considered multifactorial disorders as lifestyle and dietary behaviors can contribute to the onset and progression of dementia. The beneficial effect of exercise and diet in maintaining the function of the glymphatic system has been recently highlighted [22].

## 2. The Glymphatic System Model

**Figure 1:**
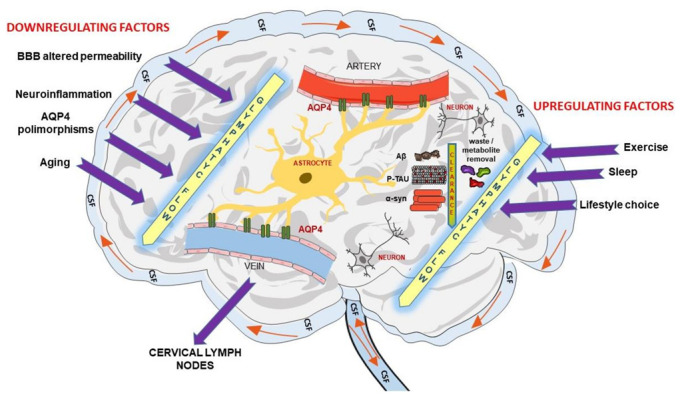
Schematic figure of the current model of the Glymphatic system.

The concept of the glymphatic system continues to evolve. Its model is currently described by a network of extravascular channels that permits the circulation of CSF and interstitial fluid within and through the brain parenchyma. This model, summarized in Figure 1, theorizes that CSF transit is a two-phase process [3,6,23,24,25,26]. First, CSF influx from the ventricular system into the subarachnoid spaces and ultimately into periarterial channels by bulk-flow—driven by arterial pulsations, inspiratory–expiratory pressure changes and CSF production [27]. From the glymphatic channels, flow is then facilitated by astroglial AQP4 into the brain interstitium, where the CSF merges with the brain extracellular fluid containing peptides and metabolites, then the fluid outflows through perivenous space or cross the dura and clear via meningeal to cervical lymphatics [5,28,29,30]. Recent studies describe macrocellular CNS clearance of fluid and metabolites through the lymphatic pathways [26]. These lymphatic vessels are located throughout the skull base and cerebral convexity dura and have distinct structural differences from their peripheral counterparts. Emerging evidence suggests that glymphatic and meningeal lymphatic structures work together [26].

In a more integrated view, fluid movements inside the cranium are the results of several compartments as clearly and elegantly described in the review of Argawal and Carare. Neurofluids (blood, CSF, and ISF) physiology takes into consideration not only the glymphatic system in the role of drainage of fluids but other waste clearance pathways such as intramural periarterial drainage pathway (IPAD), flow along cranial nerves, and meningeal lymphatics along the dural venous sinuses [1,3,31,32,33,34]. In this scenario, the fluid movements are correlated, and failure in one compartment can initiate a cascade of events affecting the clearance of waste products in the brain leading to neurodegeneration and dementia [31].

Recently, the glymphatic system has been hypothesized to facilitate the clearance of senescent cells from the brain. This report highlights functional and structural connections between the glymphatic system and extracranial lymphatic drainage pathway as well the role of this mechanism in age-related diseases such as AD [35]. In this regard, the study of Li and collaborators revealed that clearance of senescent astrocytes through meningeal lymphatics depends on the vascular endothelial growth factor C (VEGF-C)/C-C motif chemokine ligand 21 (CCL21) pathway [35].

## 3. The Glymphatic System in AD and PD

The most common neurodegenerative dementia is AD, a progressive neurodegenerative disorder affecting over 50 million people worldwide, which represents a rising challenge for public health care worldwide [36,37,38]. The disease is irreversible and presents neurodegeneration caused by toxic aggregation of extracellular amyloid plaques and intracellular neurofibrillary tangles of hyperphosphorylated tau protein [39,40]. Toxic protein accumulation causes neuronal damage, leading to cognitive decline and changes in personality and behavior [41].

Parkinson’s disease is a neurodegenerative movement disorder characterized by loss of dopaminergic neurons in the substantia nigra (SN) pars compacta and accumulation of misfolded a-synuclein in intracytoplasmic inclusions called Lewy bodies (LBs) [42]. Its characteristic motor symptoms are tremor, rigidity, bradykinesia/akinesia, and postural instability, but the clinical picture includes other motor and non-motor symptoms (NMSs) [41]. AD pathological hallmarks such as extracellular amyloid and tau aggregates are also found in patients with PD dementia and with PD-mild cognitive impairment [43,44]. Furthermore, even subthreshold amyloid might contribute to cognitive decline in patients with PD, where lower baseline CSF Aβ42 is associated with a faster rate of cognitive decline, worse performance in executive function, and delayed memory recall [45,46,47,48,49]. Therefore, it is likely that AD pathologies (beta amyloid and tau) may act synergistically with a-synuclein pathology to confer a worse prognosis [9].

The classic amyloid cascade hypothesis conceives that the accumulation of amyloid-β is the early player in AD pathogenesis and that the progression of the disease, including the formation of neurofibrillary tangles containing tau protein, results from an imbalance between Aβ production and Aβ clearance [50,51,52]. Other common neurodegenerative disorders are identified by intra and/or extracellular accumulations of a particular protein which characterizes the different neurodegenerative pathologies, such as α-synuclein in Lewy bodies and neurites in PD. Both intracellular and extracellular accumulation drive neurodegeneration and are involved in the pathogenesis of AD and PD, but understanding their biological relationship with the glymphatic system is yet to be explored.

As previously cited, impaired clearance and degradation of Aβ contribute to AD pathogenesis. The frequency of production of Aβ is estimated to be up to one molecule per second per neuron [53]. The concept that highly effective mechanisms for Aβ degradation and clearance to prevent its accumulation in the brain are required is not new [54]. In recent years, growing evidence pointing at the glymphatic system as a pathway for the peripheral clearance of solutes and proteins from the brain, including Aβ, have been collected in animal model of AD and AD patients [1,55].

Likewise, the glymphatic drainage is supposed to play a role in the removal of α- synuclein and consequently in the progression of PD. α- synuclein deposition negatively correlated with AQP4 expression in the brain of PD patients leading to the relationship between glymphatic dysfunction and protein accumulation [56].

Braak and colleagues have proposed that both Aβ and α synuclein accumulation has a recognizable pattern of spread throughout the brain and that this propagation has prion-like characteristics [57,58,59,60]. The “prion-like propagation” hypothesis has been extended to PD and AD, given the commonality of amyloid accumulations in prion disease and these neurodegenerative diseases in which cell-to-cell transmission and regional spread throughout the brain of toxic proteins seem to parallel clinical symptoms and neuropathological findings. In this regard, a drainage system like the glymphatic system acts as a conduit to facilitate the clearance and counteracts the accumulation of toxic proteins or, in the case of “conduit failure” may contribute to neurodegeneration and brain pathology [1,61]. The lymphatic system may also contribute to Aβ clearance. Following this hypothesis, Pappolla et al. have found that Aβ is present in the cervical and axillary lymph nodes of AD transgenic mice and that Aβ levels in lymph nodes increase over time, mirroring the increase of Aβ levels observed in the brain [62]. They also demonstrated that Aβ concentration was very low in other peripheral of the same animals, strongly suggesting that Aβ peptides in lymph nodes are derived from the brain [62]. The authors also suggest that biological insults that may lead to lymphatic system dysfunction may link viral infection or age-related immune dysfunction to the Aβ accumulation in sporadic AD [62].

The clearance of Aβ and tau into the CSF is the basis of the measurement of these proteins in CSF and their use as clinical biomarkers of AD [63,64]. Aβ is supposed to be transported across the blood-brain barrier (BBB). The evidence of the presence of Aβ in human lymph nodes strongly supports the idea that BBB may not be the only exit route from the central nervous system for Aβ and other proteins [65]. In recent work, Nauen and Troncoso demonstrated the presence of Aβ in human lymph nodes by analyzing the difference in the number of Aβ-labeled cells in cervical compared to inguinal lymph nodes and in this way, inferred the clearance of Aβ from the brain via the glymphatic system [65].

Aging is considered a risk factor for neurodegenerative diseases as AD and PD. Furthermore, altered circadian rhythms characterize aging, and sleep deprivation is a significant risk factor for glymphatic misfunction [27]. Aging has been also associated with a declined exchange efficiency between the subarachnoidal CSF and brain parenchyma [66]. The hypothesis that the glymphatic system declines with age is corroborated by findings in both experimental models and patients with neurological pathologies [67,68,69,70,71].

A link between circadian clock function and neurodegeneration has been recently studied by McKee and collaborators [72]. Their work investigated how astrocyte activation induced by Bmal1 deletion regulates astrocyte gene expression, Aβ plaque-associated activation, and plaque deposition [72]. The deletion of the core circadian clock gene Bmal1 abrogates clock function and induces cell-autonomous astrocyte activation [72].

## 4. Glymphatic Disfunction and Neuroinflammation

Neuroinflammation associated with AD is another factor which could exacerbate glymphatic clearance impairment [67,73]. Soluble Aβ oligomers and Aβ fibrils bind with microglia receptors leading to the release of proinflammatory cytokines [73,74,75,76,77,78]. Inflammation eventually amplifies Aβ accumulation leading to Aβ over-production and decreased Aβ clearance. Consequently, the reactive astrocytosis and changes in microglial cell morphology could cause an additional slowing of glymphatic flow [67,73,74,75,76,77,78,79] Astrogliosis is a typical tract in neurodegenerative diseases and contributes to neuroinflammation. In this regard, astrocytes from Bmal1 knockout mice crossed to the APP/ PS1-21, and the APPNL-G-F models of Aβ accumulation showed a unique transcriptional profile affecting genes involved in the generation and elimination of Aβ [72]. This astrogliosis did not affect plaque accumulation or neuronal dystrophy in either model. Astrocytes from knockout mice in this gene show enhanced activation responses to amyloid-beta [72]. Further, AQP4 expression and distribution alteration contribute to the process [80]. The review of Mogensen et al. (2021) recalls that an increase in AQP4 expression in inflammation or injury does not correspond to an increased glymphatic flow as loss of the vascular polarization of AQP4 correlates with a decrease in glymphatic flow [73]. As previously said, the loss of AQP4 vascular polarization has been described in aging and other neuropathological conditions [67,71,73,80].

It is worth noting that an impairment of the brain’s drainage system may accelerate the neuroinflammatory response. In addition to glymphatic disruption, alterations in meningeal lymphatic vessel (MVL) functions can contribute to neurological conditions such as traumatic brain injury, AD, and PD [67,73]. Recent work showed that ablation of drainage through the meningeal lymphatic vessels in a mouse AD model exacerbated amyloid-β deposition, neurovascular dysfunction, microgliosis, and behavioral deficits [67,79].

## 5. Glymphatic System and Tau Pathology in AD and Other Neurodegenerative Diseases

Tauopathies are neurodegenerative diseases characterized by a common pathological hallmark: aggregated tau deposition in the brain [81,82,83,84,85]. Aβ accumulation represents an upstream pathophysiological event and may function as a trigger/facilitator of downstream molecular pathways, including tau misfolding, tau-mediated toxicity, accumulation in tangles, and tau spreading that leads to cortical neurodegeneration [86,87,88,89,90,91]. Tau is significantly elevated in CSF of AD patients, and its increase is an early event before the onset of the clinical signs [92]. Further, both total and phosphorylated tau (p-tau) are increased in the CSF of AD patients and can predict the progression of the disease [92,93,94]. It is not clear if the increased tau in CSF, in AD or in other pathological conditions is due to the passive release of tau into the extracellular spaces by injured or dying neurons or to tau over-production and/or tau decreased clearance [95,96,97]. Even if the accumulation of Aβ has been considered the primary injury and therapeutic approaches have been targeted towards Aβ removal, the subsequent tau pathology and tau-mediated neurodegeneration suggested that tau pathology can progress independently of Aβ accumulation [97]. Tau-targeted therapies in alternative to Aβ-targeted treatments have recently emerged as potential strategies for treating AD patients [98,99].

Recently, a tau interactome study revealed tau interactions with presynaptic vesicle proteins during activity-dependent tau secretion and mapped the tau-binding sites to the cytosolic domains of integral synaptic vesicle proteins [100]. Tracy and collaborators (2022) showed that MAPT mutations impair bioenergetics and markedly diminished tau’s interaction with mitochondria proteins, downregulated in AD brains of multiple cohorts and correlated with disease severity. This study highlights potential therapeutic targets to block tau-mediated pathogenesis in neurodegenerative diseases [100].

The levels of tau in the ISF are, as well as Aβ, the result of the balance between their release in the extracellular space and their clearance. The intriguing hypothesis of the glymphatic system’s involvement and therapeutic potential in extracellular tau clearance has been recently studied [61,101,102,103]. Tau is not only an intracellular protein but can be secreted in the extracellular space, and it is the basis of the hypothesis that the glymphatic system can contribute to the spread of tau pathology to anatomically related areas [104,105]. Evidence suggests that the glymphatic system, clearing the extracellular space, acts as a conduit for neuron-to-neuron propagation and regional progression of AD tau pathology [106,107,108,109].

In a recent study, significantly higher levels of AQP4 were found in AD and FTD patients compared to subjects not affected by neurodegenerative diseases, and a significant, positive correlation between AQP4 and total Tau levels was found. Authors discussed the link between glymphatic system alteration and neurodegeneration with clinical and molecular evidence [110].

## 6. AQP4 Expression and Polymorphisms in AD and PD

As previously pointed out, the glymphatic clearance is based on the astrocytic AQP4-dependent flow that facilitates the clearance rate of exogenous tracers primarily during sleep when the flow clearance is enhanced by more than double [111,112]. AQP4 deficiency has been shown to reduce Aβ clearance and to influence amyloid deposition and neuronal functions in mice [113,114,115,116]. In aging human brains, a postmortem study revealed a link between reduced perivascular localization of AQP4 and increased Aβ deposition [117].

Ishida and collaborators have recently shown that deletion of AQP4 in the brains of transgenic mice expressing P301S mutant tau not only elevated tau in CSF but also markedly exacerbated p-tau deposition and the associated neurodegeneration [109]. The study suggests that impairment of glymphatic clearance of extracellular tau is a regulatory mechanism which contributes to tau aggregation and neurodegeneration [109]. The authors also discuss the possibility that AQP4 deficiency exacerbated tau aggregation creating a vicious cycle between impaired glymphatic clearance and tau aggregation [109]. Even though the mechanisms of how the impairment in clearing extracellular tau led to the exacerbation of tau-related pathology are unclear, the authors suggest that the impairment of tau clearance in AQP4-deficient PS19 mice promotes the spreading of pathological tau species to other cells [107]. In conclusion, deletion of AQP4 or pharmacological inhibition of AQP4 exacerbates pathogenic accumulation of Aβ and tau in AD transgenic mouse models [109].

Recent genetic studies revealed that Single Nucleotide Polymorphisms (SNPs) of the AQP4 gene were associated with altered rates of cognitive decline after AD diagnosis, with two SNPS (rs9951307 and rs3875089) associated with slower cognitive decline and two (rs3763040 and rs3763043) associated with more rapid cognitive decline after AD diagnosis. AQP4 genetic variation was associated with Aβ accumulation, disease stage progression, and cognitive decline and could be considered a useful potential biomarker in predicting disease burden for those in the spectrum of AD [118,119]. AQP4 SNPs is also associated with reduced perivascular AQP4 localization in AD patients [118,119].

Regarding PD patients, Fang and collaborators have recently conducted a study to determine the clinical implication of AQP4 polymorphisms in PD [120]. They investigated whether AQP4 SNPs were associated with Aβ burden as measured by 18F Florbetapir standard uptake values and examined if AQP4 SNPs moderated the association between REM sleep behavior disorder (RBD) and CSF biomarkers [120]. They conclude that genetic variations of AQP4 and subsequent alterations of glymphatic efficacy might contribute to an altered rate of cognitive decline in PD [120]. Furthermore, AQP4 rs162009 can be considered a novel genetic prognostic marker of glymphatic function and cognitive decline in PD [120].

## 7. The Glymphatic System in Neuroimaging Studies

A non-invasive method to measure glymphatic pathway function may be considered an early approach in asymptomatic or diagnosed patients to evaluate disease susceptibility and progression. Dilated perivascular spaces observed by magnetic resonance imaging (MRI) can be used as a biomarker of glymphatic dysfunction and amyloid accumulation in AD and other neurological diseases [121]. MRI of the glymphatic system is performed using intrathecal injections of gadolinium-based contrast agents has been used in humans to visualize the glymphatic system. However, the administration of gadolinium-based contrast agents could lead to severe neurotoxic complications [122,123]. Taoka and collaborators (2017) have proposed a non-invasive measurement method, diffusion tensor imaging along the perivascular space (DTI-ALPS), which is now widely used in studies on the glymphatic system of the human brain [124]. They demonstrated that ALPS index significantly negatively correlated with the Mini-Mental State Exam score in relation to AD severity [124]. A damaged glymphatic system, evaluated by DTI-ALPS, has also been demonstrated in other studies and in patients with different pathologies [125]. McKnight et al. (2021) reported that the ALPS index in PD patients was also significantly lower than that in patients with essential tremor and supposed that may be related to changes in the glymphatic transport system due to abnormal protein aggregation in PD [126]. Furthermore, they found correlations between the ALPS index and age and T2-weighted white matter hyperintensity [126]. In a follow-up study, they assessed the correlation between the ALPS index and the progression of PD. Interesting observational research by Si et al. (2022) demonstrated a sequential decrease in the ALPS index from prodromal PD to clinical PD [127]. Further, the ALPS index was related to disease severity in patients with sleep behavior disorders and patients with PD [127]. As the authors stated, the study lacks an intervention to modify the glymphatic system, and further experimental evidence is needed to confirm that DTI-ALPS measures glymphatic function [127]. DTI-ALPS evaluation has also been used to study glymphatic system dysfunction in patients with hemorrhagic stroke, where DTI-ALPS index reflected disease duration [128]. These findings demonstrate the importance of DTI-ALPS in detecting functional changes in the glymphatic system and underscore the potential value of the ALPS index as a biological indicator of neuropathological conditions.

In addition to glymphatic disruption, MVLs functions can contribute to many clinical conditions such as traumatic brain injury, AD, multiple sclerosis, and PD [13,129,130,131,132]. Albayram and collaborators (2022) have recently proposed a non-invasive, non-contrast 3D fluid-attenuated inversion recovery (FLAIR) MR method permitting detailed visualization of dorsal—along the venous sinuses and ventral MLVs—around perineural/peridural spaces of cranial nerves, CSF/ISF drainage around nerves in the human brain, as well as visualization of direct relationships among these pathways and deep cervical lymph nodes [13].

The use of PET studies and neuroimaging has also shed some light on the physiology of the glymphatic system and its role in clearing the human brain [133]. Li and collaborators recently studied the relationship between brain Aβ deposition and its impaired clearance in sporadic AD using a PET study [133]. This PET study measured CSF clearance and the amyloid burden and used T1-weighted MRI to estimate brain atrophy in mild AD and healthy elderly participants [133]. Their findings support the hypothesis that failed CSF clearance is characteristic of AD and related to Aβ deposition [133]. The authors underscore the need for further longitudinal studies to determine whether impaired CSF clearance predicts progressive amyloidosis or its consequence [133].

Using neuroimaging, Zou et al. (2019) assessed glymphatic dysfunction in an animal PD model [134]. They blocked the meningeal lymphatic vessels in A53T mice and observed α-syn deposition six weeks later, accompanied by motor dysfunction [134]. This finding strongly suggested that the glymphatic system dysfunction aggravates the accumulation of α-synuclein and further accelerates the disease progression of PD [134]. Given the limited evidence supporting the association between glymphatic system malfunction and α-synucleinopathy in humans, it seems crucial to identify a neuroimaging marker to detect glymphatic system changes in patients with PD.

Harrison and collaborators have studied the glymphatic system using MRI in a mouse model that develops tau NFT pathology [61]. In this model, they traced the clearance of parenchymal tau using intracerebral injections and CSF sampling and studied the modulation of AQP4 function [61]. By using contrast-enhanced MRI, they provided a spatial and temporal description of the glymphatic system in the mouse brain, highlighting the critical role of this clearance system in the deposition of tau protein in the brain [61].

## 8. Conclusions

Even if neurofluid drainage function of the brain is an integrated system based on different compartments, the glymphatic system and AQP4 could be considered intervention targets in neurodegenerative diseases. The glymphatic system’s increased function and efficiency could contribute to preventing or delaying the accumulation of proteins in the brain. In this scenario, the glymphatic system could also be necessary as a player in the clearance of tau, and special attention to Aβ independent regulators of tau, such as the glymphatic system, should be deserved in the study of neurodegenerative tauopathies.

Given the importance of impaired molecular clearance from CSF to blood in the development of neurological diseases, the direct measurement of CSF to blood clearance on an individual basis has been studied for paving the way toward personalized intrathecal drug administration in CNS [135].

A therapeutic intervention to modify the glymphatic system is not known; behavioral or pharmacological interventions that preserve night sleep could enhance glymphatic function, especially in the early stages of AD when the drainage system is still intact [79]. To this regard, the lack of the expected benefits of antibody-based therapies, especially in the advanced stage of disease or in the advanced age, could be explained by a declined function of the glymphatic system [79].

The study of AQP4 polymorphisms has amplified the knowledge of genetic predisposition to neurodegenerative diseases and underscored the association of AQP4 polymorphisms with cognitive performance in AD and PD in the pathophysiology of these diseases. Regarding the role of AQP4 in glymphatic system dysregulation, it is worth noting that there are very few studies on the manipulation of the glymphatic system through AQP4 inhibitors or neuroprotective agents.

Finally, it seems crucial to identify a neuroimaging marker to detect glymphatic system changes. In this regard, non-invasive methods can be considered tools to detect glymphatic dysfunction and be used as a new potential biomarker in AD and PD.

## Data Availability

Not applicable.
